# Hypertension in chronic kidney disease: What lies behind the scene

**DOI:** 10.3389/fphar.2022.949260

**Published:** 2022-10-11

**Authors:** Omar Z. Ameer

**Affiliations:** ^1^ Department of Pharmaceutical Sciences, College of Pharmacy, Alfaisal University, Riyadh, Saudi Arabia; ^2^ Department of Biomedical Sciences, Faculty of Medicine, Macquarie University, Sydney, NSW, Australia

**Keywords:** sympathetic hyperactivity, renin-angiotensin-aldosterone system, vascular remodeling, endothelial dysfunction, chronic kidney disease

## Abstract

Hypertension is a frequent condition encountered during kidney disease development and a leading cause in its progression. Hallmark factors contributing to hypertension constitute a complexity of events that progress chronic kidney disease (CKD) into end-stage renal disease (ESRD). Multiple crosstalk mechanisms are involved in sustaining the inevitable high blood pressure (BP) state in CKD, and these play an important role in the pathogenesis of increased cardiovascular (CV) events associated with CKD. The present review discusses relevant contributory mechanisms underpinning the promotion of hypertension and their consequent eventuation to renal damage and CV disease. In particular, salt and volume expansion, sympathetic nervous system (SNS) hyperactivity, upregulated renin–angiotensin–aldosterone system (RAAS), oxidative stress, vascular remodeling, endothelial dysfunction, and a range of mediators and signaling molecules which are thought to play a role in this concert of events are emphasized. As the control of high BP *via* therapeutic interventions can represent the key strategy to not only reduce BP but also the CV burden in kidney disease, evidence for major strategic pathways that can alleviate the progression of hypertensive kidney disease are highlighted. This review provides a particular focus on the impact of RAAS antagonists, renal nerve denervation, baroreflex stimulation, and other modalities affecting BP in the context of CKD, to provide interesting perspectives on the management of hypertensive nephropathy and associated CV comorbidities.

## 1 Introduction

Chronic kidney disease (CKD) is the most common cause of secondary hypertension (Campese et al., 2006; Masuda and Nagata, 2020), in which hypertension is considered an important co-morbid factor associated with CKD (Baumeister et al., 2009; Middleton and Pun, 2010), and is the leading cause of death globally (Unger et al., 2020; Guan et al., 2022). Hypertension presents in approximately 80–85% of patients with CKD (Wong et al., 2006; Collins et al., 2014; Freedman and Cohen, 2016), with the more severe glomerular diseases having a higher incidence of hypertension (Blythe, 1985; Sanchez et al., 2018). For any given cause of CKD including hypertension itself, the elevation in systemic blood pressure (BP) accentuates the rate at which glomerular filtration rate (GFR) declines (Perry et al., 1995; Ku et al., 2019), which makes hypertension an independent risk factor for end-stage renal disease (ESRD) (Tozawa et al., 2003; Salman, 2015). Previous reports have noted that hypertension increases the risk for new or recurrent cardiovascular (CV) events in individuals with stage 2–3 CKD (Muntner et al., 2005). This is also evident for genetically driven causes of CKD, with virtually all patients with polycystic kidney disease (PKD) for example, developing hypertension by the time ESRD is present (Chapman et al., 2010). Reports suggest that the high BP state is considered a strong independent risk factor for ESRD and interventions to prevent the disease need to emphasize the prevention and control of both high-normal and high BP (Klag et al., 1996; Ku et al., 2019). Hypertension is therefore associated with accelerated progression of kidney disease as well as the development and worsening of cardiovascular disease (CVD).

The kidney has an intrinsic role in the development of hypertension. There is both clinical and experimental evidence to suggest that the “*BP goes with the kidney*” (Adamczak et al., 2002). Indeed, it has been shown that normotensive recipients of kidneys from hypertensive donors become hypertensive in humans (Guidi et al., 1996) and in rats (Rettig et al., 1990); and conversely hypertensive recipients of normotensive kidneys may become normotensive again in both humans (Curtis et al., 1983) and rats (Patschan et al., 1997). In addition, risk factors associated with BP elevation and its development are similar to those in the general population after kidney transplantation (Sanchez et al., 2018). Presently, it remains somewhat vague as to what is the best target BP in the treatment of hypertensive patients with CV risks. As such, this is creating a grey area in the selection between reducing the risk of CV incidence and mortality, or risking having poor prognostic or damaging effects on the kidneys themselves. In the Systolic BP Intervention Trial (SPRINT) study (Group, 2015), aggressive BP reduction with antihypertensives to target a systolic BP of <120 mmHg (relative to the standard target of systolic BP < 140 mmHg) has been shown to reduce the rate of CV risks (myocardial infarction, acute coronary syndrome, heart failure, stroke, or CV deaths) or all-cause mortality. An unfortunate association noted with this outcome, however, was a higher risk of renal injury in the intensive BP lowering cohorts (Obi et al., 2018; Rocco et al., 2018), or failure to produce a benefit for kidney GFR decline or ESRD risk (Schrier et al., 2014; Cheung et al., 2017). Regardless of CKD etiology, severe and uncontrolled hypertension accelerates the loss of nephrons, and hence GFR (Ku et al., 2019). However, whether intensive BP reduction slows GFR decline is evidently still unclear. Furthermore, the disagreement on the cut-off value for hypertension treatment guidelines in adults between Americans (target above 130/80 mmHg) and Europeans (target above 140/90 mmHg or above 130/80 mmHg with a high CV risk) (Antza et al., 2021) creates a lot of confusion for clinicians, suggesting that a large number of patients remain without BP treatment. The clinical scenario around elevated BP with continual monitoring should be taken into careful consideration, and a decision around lowering BP treatment should always be carried out while placing the utmost priority on patient’s outcomes. It is important to critically decide whether long-term treatment goals should have CV or renal protection benefits, as achieving a compatible target BP in order to balance the cardio-renal protection axis has proved challenging. Of importance to note is that the kidneys may play a key “*cause and effect*” role: patients with CKD have an elevated CV risk, and hypertension is one of the leading risk factors for CV events and stroke (Townsend and Taler, 2015), and is known to worsen renal impairment.

## 2 Pathogenesis of hypertension in chronic kidney disease

**FIGURE 1:**
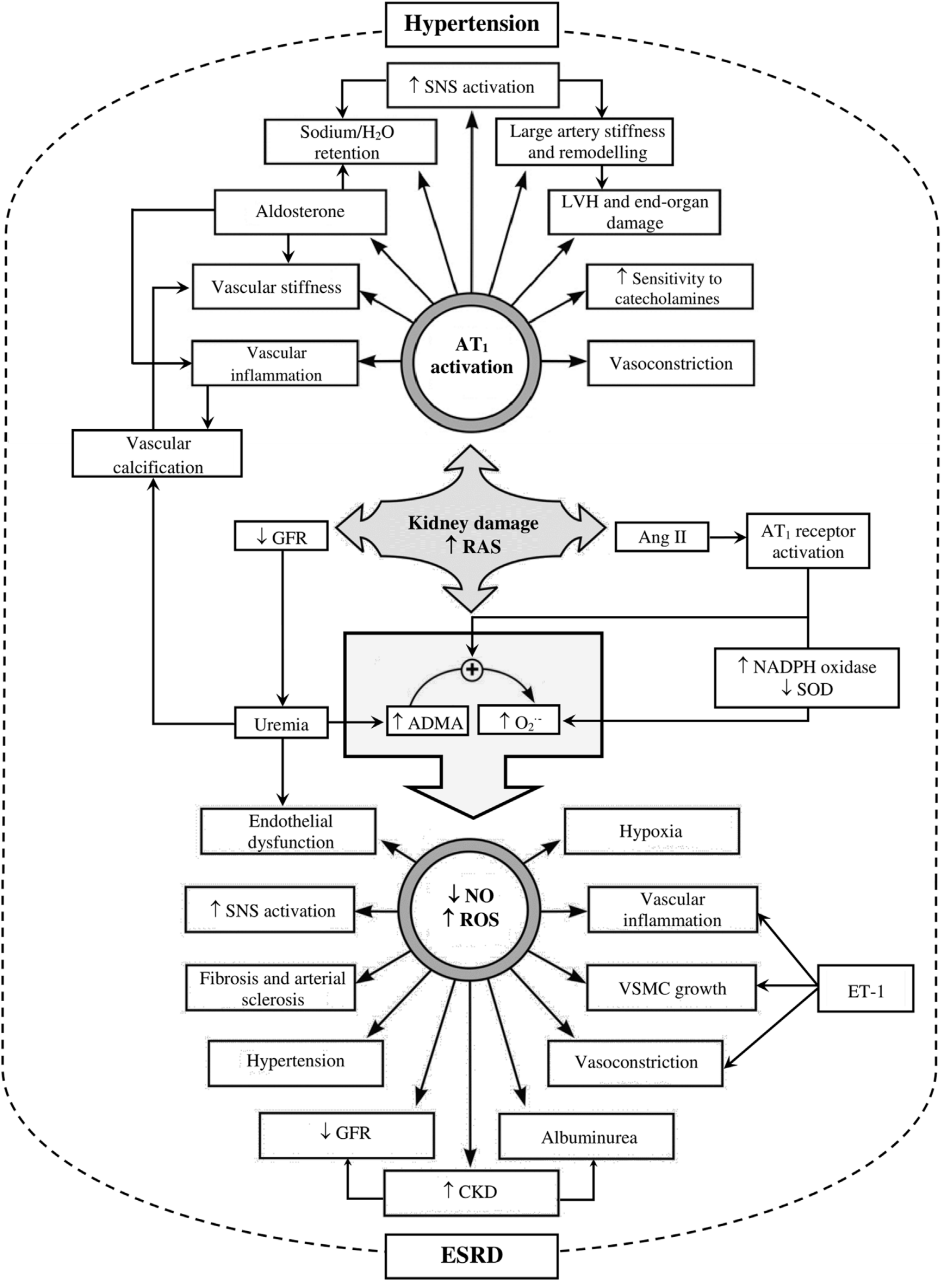
Schematic representation of factors involved in kidney damage, hypertension and end-stage renal disease (ESRD). Figure shows complications arise upon activation of the renin-angiotensin system (RAS) secondary to kidney damage. Increased RAS activity subsequently activates angiotensin-1 receptor (AT_1_ receptors) via increasing angiotensin II (Ang II) and decreases glomerular filtration rate (GFR). Angiotensin 1 receptor activation results in a myriad of effects such as vascular remodeling and inflammation, aldosterone release, sodium/H_2_O retention, disturbs the balance between antioxidant (superoxide dismutase; SOD) and oxidant (nicotine amide dinucleotide phosphate; NADPH) mechanisms, and increase sympathetic nervous system (SNS) activation. A reduction in GFR causes uremia, which is associated with increased asymmetric dimethylarginine (ADMA) and oxidative stress, all of which predispose to endothelial dysfunction and loss of nitric oxide (NO) functional signaling. Impairment of the NO pathway and increased reactive oxygen species (ROS) can subsequently predispose to the aggravation of SNS activation, vascular remodeling, and inflammation, further elevating systemic hypertension. Endothelin *via* endothelin-1 receptors (ET-1), on the other hand, can directly contribute to vasoconstriction and vascular inflammation and hypertrophy. In addition, CKD can also trigger vascular calcification, which is a hallmark of cardiovascular (CV) complications associated with the disease state. Together, these factors can consequently trigger/enhance the development of CKD complications and progression to ESRD. LVH, left ventricular hypertrophy; O_2_^•-^, superoxide free radical; VSMC; vascular smooth muscle cell. Figure adapted and modified from: (Palm et al., 2007; Grassi et al., 2010; Nauli et al., 2011; Briet and Schiffrin, 2013; Raina et al., 2020).

The pathogenesis of hypertension in patients with CKD is complex and multifactorial, and often resistant to treatment due to its complexity (Campese et al., 2006; Townsend and Taler, 2015; Hamrahian, 2022). Several putative mechanisms contribute to elevated BP in this disease, including neural and hormonal changes that often act in concert to disrupt appropriate BP regulation. The majority of these factors that contribute to the development of hypertension in CKD are described here (Figure 1).

### 2.1 Salt retention and volume expansion

Sodium retention is of primary importance, as under the influence of Ang II and aldosterone (RAS section in Figure 1), which both promote sodium reabsorption in the proximal and collecting tubules respectively (Rodriguez-Iturbe et al., 2007), there can be a total loss of GFR in renal disease. This impairs sodium excretion, and therefore leads to further sodium retention and extracellular expansion (Blaustein et al., 2006; Ku et al., 2019). Even though the degree of extracellular volume expansion may be insufficient to induce edema, hypertension can still be evoked (Vertes et al., 1969; Preston et al., 1996). Sodium retention causes hypertension through volume-dependent and volume-independent mechanisms (Sica and Carl, 2005). An excess extracellular volume (volume-dependent) leads to increased perfusion of peripheral tissues, which stimulates vasoconstriction, heightening peripheral vascular resistance, and consequently rising BP (Dal Canton et al., 1996; Sica and Carl, 2005). This latter effect is ultimately due to the direct volume expansion, and indirect production of endogenous cardiotonic steroids or ouabain-like steroids (Schoner, 2002), which may further favor an increase in sodium load, BP and vascular remodeling (Blaustein et al., 2009). On the other hand, volume-independent mechanisms include increased vascular stiffness and increased central SNS outflow as a result of excessive sodium retention (Saxena et al., 2018) (SNS overactivity section in Figure 1). In addition to the aforementioned, excess sodium chloride load within the circulation also contributes to the autoregulation phenomena (Salem, 2002) (Figure 1). Physiologically, this autoregulation mechanism shields the capillary tuft of the glomeruli within nephrons from the elevated systemic BP. Thus, the afferent arteriole myogenic diameter changes in response to systemic BP reflexively, and sodium chloride delivery to the macula densa exerts a tubulo-glomerular feedback to maintain the intraglomerular pressure, and hence maintain a normal GFR. However, under the pathological state of chronic systemic BP elevation, the high pulsatility remodels and/or stiffens the afferent arteriole and reduces its ability to constrict and dilate. This subsequently leads to glomerular hypertension, nephrosclerosis, and progressive deterioration of kidney function. It is noteworthy to mention here with respect to certain medications like non-steroidal anti-inflammatory drugs (NSAIDs) such as indomethacin or ibuprofen, perturbs the afferent arteriole blood flow to the nephron via inhibition of the vasodilating PGI_2_ cyclooxygenase (COX) product (Baker and Perazella, 2020), and hence patients taking these drugs chronically can inadvertently accelerate kidney damage and impair the glomerulus shielding effect.

Alterations in vasoactive substances or abnormal ion channel function may play a role in the development of hypertension in ESRD (Martinez-Maldonado, 1998). For example, we know that sodium chloride intake potentiates the pressor action of endogenous catecholamines, such as norepinephrine (NE) (Ito et al., 1989) (Figure 1). An overt sodium intake results in stiffer blood vessels, vascular inflammatory changes, and loss in nitric oxide (NO) functionality (Figure 1); processes that are known to contribute to BP elevation (Hovater and Sanders, 2012). In the face of fluid and salt overload, in accordance with research strictly reducing sodium intake to < 1.5 g/d (Ku et al., 2019) is recommended, and that this should be the earliest non-pharmacological intervention to initiate (Ku et al., 2019; Masuda and Nagata, 2020). Such management strategies can be more robust at the start of a confirmed BP elevation, because with time, some patients can become resistant to sodium-mediated changing maneuvers, by showing no discernible response in their BP when challenged by low and high sodium diets. The ability to maintain a normal BP state in the face of a sodium challenge may be hampered with progressive loss in kidney function, and a decline in GFR is aligned with a greater sensitivity of BP to salt (Koomans et al., 1982).

In view of the above, it can be concluded that salt retention can compromise CV regulation and negatively impact disease progression in CKD via both a direct and indirect actions, whereby salt expands plasma volume and alter regulation of vasoactive and hormonal substances key to BP control, respectively.

### 2.2 Sympathetic nervous system activity

Autonomic neuroregulation of CV function which governs periodic fluctuations in heart rate and BP, depends on a balance between the sympathetic and parasympathetic activity to the heart and vasculature (Salman, 2015). In CKD, a state of functional disharmony between sympathetic and parasympathetic arms of the autonomic nervous system, with altered tonic and reflex control of autonomic outflows, is seem, culminating in autonomic dysfunction and SNS hyperactivity (Salman, 2015). Overactivity of the SNS augments the high BP state via an increase in cardiac output and total peripheral resistance. This may result from direct actions on cardiac and vascular receptors, or by the renin-angiotensin system’s (RAS) influence on the release of renin and sodium retention by the kidney (Martinez-Maldonado, 1998). This latter mechanism may thus contribute to target organ damage and adverse clinical outcomes, independent of BP (Kelm et al., 1996; Mancia et al., 1999; Greenwood et al., 2001) (Figure 1).

The enhanced SNS activity has been demonstrated in patients with CKD and ESRD (Campese and Krol, 2002; Neumann et al., 2004; Blankestijn, 2007; Vink et al., 2013; Sata et al., 2018). In addition to showing increased muscle sympathetic nerve activity (SNA) (Converse et al., 1992; Johansson et al., 1999; Neumann et al., 2004), renal failure patients also demonstrate increased circulating plasma catecholamines, as measured by plasma levels or total body NE spillover (Cerasola et al., 1998; Johansson et al., 1999; Zoccali et al., 2002; Klein et al., 2003a). Supporting this SNS overactivity is the evidence that administration of debrisoquin, a post-ganglionic sympathetic blocker, significantly reduces BP in hypertensive dialysis patients, but not in controls or normotensive dialysis patients (Schohn et al., 1985). The kidneys have a dual role in the pathogenic rise of sympathetic activity as they are both a recipient of efferent sympathetic signals as well as a generator of renal afferent activity (Vink et al., 2013; Sata et al., 2018). Stimulation of the renal efferent nerves results in a cascade of actions in the kidneys, and because of renal vasoconstriction, renal blood flow and GFR decrease, salt retention increases, and Ang II is produced. Here, the stimulation of Ang II is of particular importance, because Ang II directly causes vasoconstriction, has trophic effects, and as mentioned earlier, plays an important role in water and sodium reabsorption in the proximal tubule (Reid, 1992; Rodriguez-Iturbe et al., 2007) (Figure 1). The afferent signal in failing kidneys is also thought to contribute to an increase in BP. This is evidenced by correction of both BP and muscle SNA in patients who have undergone bilateral nephrectomy (Converse et al., 1992; Adamczak et al., 2002; Hausberg et al., 2002), and attenuation of elevated BP in animal models of renal failure, where dorsal rhizotomy (a procedure that selectively destroys the renal afferents), or renal denervation was performed (Ye et al., 1998; Campese and Krol, 2002; Ciriello and de Oliveira, 2002). Some might argue that the greater SNA in CKD might be a renal-specific response or due to a generalized stimulation of SNS activity. A study performed recently via electro-lesioning 5/6th of the cortex (glomeruli layer) of one kidney on New Zealand rabbits (Sata et al., 2020) compared renal sympathetic activity and NE spillover to those of sham control. Interestingly, their results suggested that nerve overactivity and global SNS activity originate from the kidneys, because circulating NE measurements were not the driver of increased BP in this model (Sata et al., 2020). These aforementioned results suggest that perhaps renal denervation may present a more effective treatment approach than the use of sympatholytic drugs in the context of systemic hypertension in CKD.

The relationship between increased SNS activity relative to vasculature can also be complex. As in the presence of SNS overactivity, the vasculature can become hypertrophied (Bevan, 1984), which in itself or as mediated by a complex interaction with the RAS can sensitize the vasculature to sympathetic vasomotor input (Adams et al., 1990; Smid et al., 1995); a phenomenon termed as the “*vascular amplifier effect*”*.* We have previously addressed this issue in our rat model of chronic polycystic kidney disease (PKD), the Lewis polycystic kidney disease (LPK), which has an autosomal recessive variant of PKD (McCooke et al., 2012). In this study, we evaluated the extent of the relative contribution of the SNS versus the vasculature in the maintenance of hypertension in the LPK, its normotensive control (Lewis rat) and the spontaneously hypertensive rat (SHR) as a high BP rodent model (Ameer et al., 2014a). Our data revealed that despite the presence of exaggerated depressor effects in BP to direct vasodilators (response amplification) in the CKD model, the SNS overactivity as assessed by ganglionic blockade with hexamethonium still accounted for a larger depressor effect relative to that of direct vasodilation. This was further confirmed by our BP spectral analysis of Mayer’s waves (low frequency power: range 0.2–0.6 and 0.4 Hz in rats), reflecting SNS vasomotor tone (Stauss, 2007). Indeed, relative to both the normotensive and hypertensive controls, the LPK demonstrated the steepest relationship when BP responses to ganglionic blockade were correlated with the low frequency oscillation in systolic BP (Ameer et al., 2014a). When directly recorded under both anesthetized (Salman et al., 2014; Salman et al., 2015a) and conscious (Salman et al., 2015b) conditions, baseline renal SNA was consistently higher in the LPK model of CKD compared to controls. Together, our data provide both direct and indirect experimental evidence of heightened sympathetic drive in CKD, and its potential contribution to the temporal decline in renal and hemodynamic function observed in this condition.

In experimental animals it has been demonstrated that ischemic metabolites or uremic toxins can evoke reflex increases in SNS activity and BP (Katholi, 1985), and that chronic stimulation of afferent nerves may lead to SNS overactivity and therefore an increase in BP too. Supporting this finding, an earlier study showed that uremic toxins have a stimulatory effect on SNS activity (Recordati et al., 1981). However, in contrast, other reports have shown ongoing increased SNA in patients with renal grafts, despite correction of uremia (Hausberg et al., 2002). These observations suggest that increased SNA appears to be driven by signals arising in the native kidneys that are perhaps independent of circulating uremia-related toxins.

**FIGURE 2:**
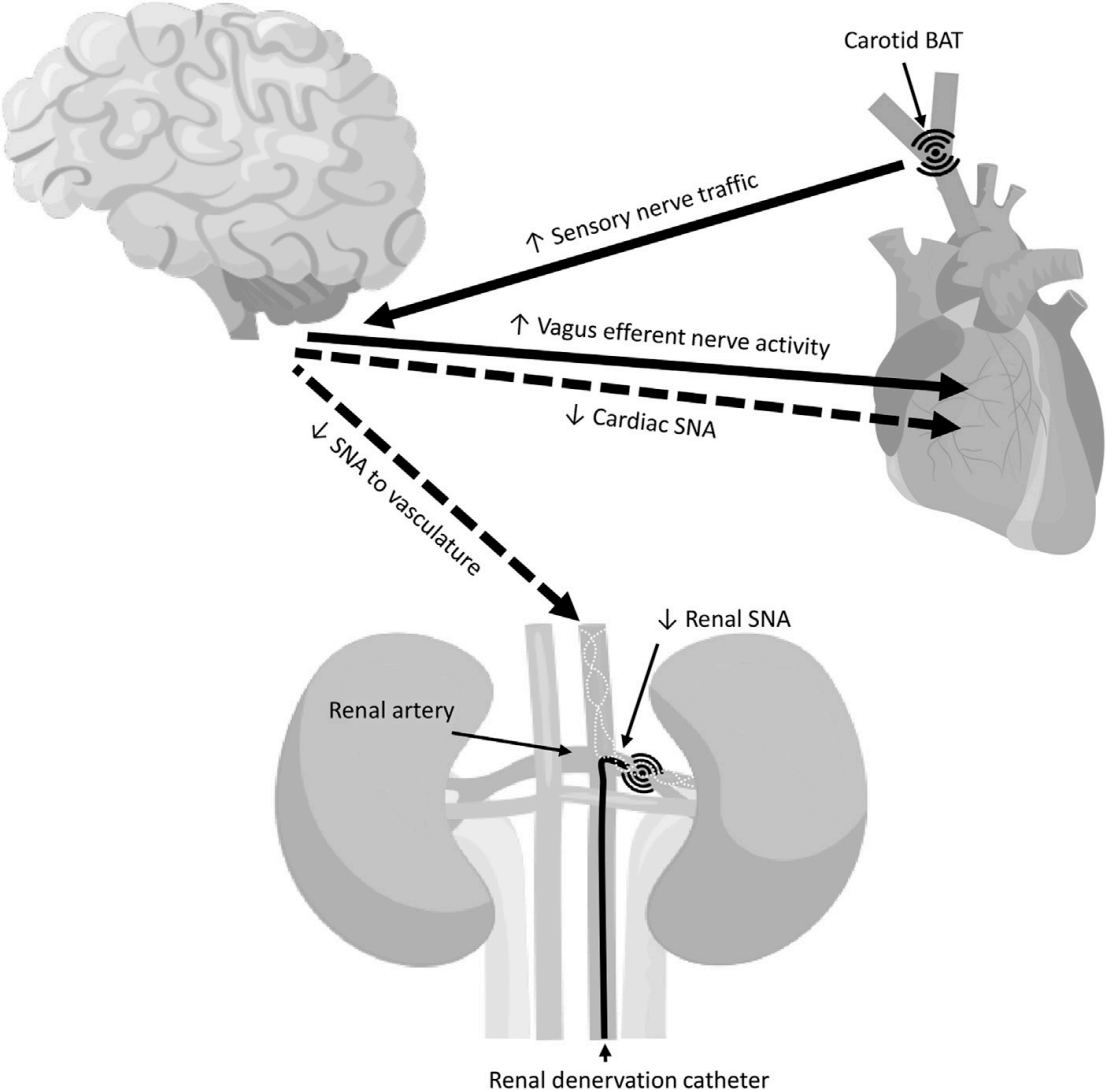
Surgical interventions to lower blood pressure (BP) in resistant hypertensive patients including baroreflex activation therapy (BAT) of the carotid baroreceptors and renal denervation using radiofrequency/ultrasound energy ablation of the renal nerves. Note that BAT evokes field stimulation of the baroreceptors within the carotid sinus, which increases sensory afferent traffic carried by the carotid sinus nerves to the brain. This results in reflex increases in vagal efferent outflow to the heart and decreases in sympathetic discharge to the heart and vasculature thereby reducing cardiac output and systemic vascular resistance, and ultimately BP. In renal denervation, on the other hand, the procedure eliminates both renal afferent and efferent activity including renal sympathetic nerve activity (SNA), the excessive activation of which is thought to be the driver of increased BP in hypertensive individuals. Adapted and modified from (Bhatt et al., 2019; Gajulapalli et al., 2020).

In addition to the above, denervation of renal nerves around the renal artery have become an important strategy in the treatment of resistant or refractory hypertension among CKD and non-CKD patients (Burnier, 2022). The procedure itself (Figure 2) involves the use of catheter-based devices that ablate the renal afferent and efferent (sympathetic) nerves surrounding the renal arteries using either radiofrequency, ultrasound energy, or trans-arterial injection of caustic substances (Gajulapalli et al., 2020). Several trials have been conducted (Krum et al., 2009; Investigators, 2010; Esler et al., 2012; Kandzari et al., 2012; Bakris et al., 2014; Bhatt et al., 2014; Liu et al., 2017; Sievert et al., 2017; Böhm et al., 2020), however to date, there is no global census or legislative approval of renal denervation as a clinical procedure for the treatment of hypertension non-pharmacologically. Although renal denervation has played an ameliorating role in reducing elevated BP and preserving renal function in diseased kidneys in humans (Hering et al., 2012; Ott et al., 2015) and rats (Yao et al., 2017) with CKD, other factors contributing to BP elevation remain, that are unalterable by renal denervation. For example, in our studies, we have shown that despite a reduction in BP and urinary protein of our CKD rat model, renal denervation did not fix the problem of increased arterial stiffness as assessed by pulse wave velocity (PWV), a hallmark of BP derived risk factor for morbidity (Yao et al., 2017). To date, the renal denervation field is challenged by: 1) a lack of trained personnel and professionals performing the procedure; 2) an unavailability of strict requirements for operation specifications; 3) nil available methods for measuring the effectiveness of denervation procedures; 4) a lack of definition with regards to which patients are optimal candidates for the denervation; 5) reported controversy in clinical outcomes, especially in relation to office or ambulatory BP readings, and GFR protection versus worsening; 6) the extent of the denervation process required; 7) nerve anatomical variations among patients; 8) the issue of re-denervation or renal nerves convergence (Yao et al., 2017) after a period of time following the procedure; 9) variation in timing and follow-up during the trials (for example, 2–36 months); (10) the presence of good representative sham-controls; and 11) the complications that arise following the procedures, which may range from minor adverse effects, to hematoma, renal artery narrowing or stenosis or dissection, acute renal failure, or even death (Sata et al., 2018; Ku et al., 2019; Liang et al., 2020). In view of the above, evidently a lot of factors need to be considered in renal denervation procedures, involving not just comparisons between clinical outcomes, but also cost effectiveness considerations between the denervation procedures and currently approved pharmacological therapy.

The participation of the SNS became more complex with the discovery of renalase oxidase, an amine oxidase enzyme (flavoprotein oxidase) involved in the regulation of cardiac function, BP and kidney injury (Xu et al., 2005; Wang et al., 2014; Czubilińska-Łada et al., 2020). It metabolizes catecholamines and is mainly expressed in the glomeruli and proximal tubules of the kidney, cardiomyocytes and other tissues (Xu et al., 2005; Wang et al., 2014). Renalase is secreted into the plasma and urine of healthy persons, yet is undetectable in uremic individuals. Reports have shown that the renalase knockout mouse is hypertensive and exquisitely sensitive to cardiac ischemia (Wu et al., 2010), and develops renal tubular damage that can be ameliorated with recombinant renalase treatment (Lee et al., 2013). The administration of recombinant renalase produces a powerful and rapid hypotensive effect on rats (Schiffrin et al., 2007; Wang et al., 2017) and reduces plasma NE, left ventricular hypertrophy (LVH), and cardiac fibrosis in 5/6 nephrectomy hypertensive CKD rats (Baraka and El Ghotny, 2012). Blood renalase levels are inversely correlated with GFR and are markedly reduced in ESRD patients (Desir, 2009). Thus, SNS overactivity could be partly associated with abnormalities in the renalase pathway in CKD, as renalase deficiency may contribute to the heightened sympathetic tone observed in ESRD patients (Li et al., 2008; Desir and Peixoto, 2014). Indeed, extended research into the various pathways and mechanisms involved in this crucial enzyme activity would no doubt result in influential outcomes in tackling conditions such as high BP and kidney disease, especially when it comes to the use of this oxidase or its analogues as a therapeutic or diagnostic tool.

As previously reviewed (Salman, 2015), a major mechanism long thought to underlie altered elevated SNS activity and therefore BP in CKD is reduced arterial baroreflex function. In hypertension, it is hypothesized that the baroreceptor sensitivity is reset to a higher operating pressure. Possible explanations for this phenomenon are direct damage to the receptors, a change in the coupling between receptors and vascular walls, genetically determined properties of the receptors, and decreased distensibility of the vascular walls in which receptors are embedded (Salman, 2015). Heart rate baroreflex sensitivity, either spontaneous (Lacy et al., 2006; Studinger et al., 2006; Johansson et al., 2007) or evoked by pharmacologic manipulation of BP (Agarwal et al., 1991; Tinucci et al., 2001; Studinger et al., 2006), are blunted in CKD, and most importantly, deficits show a strong correlation with reductions in GFR (Lacy et al., 2006). This therefore suggests a direct association between deterioration in renal function and alterations in the cardiac baroreflex, rendering these markers useful in determining CV risk in CKD. In contrast to clinical studies investigating heart rate baroreflex, those directly assessing sympathetic baroreflex in CKD patients are conflicting, perhaps due to the limited number of reports directly assessing SNA baroreflex function in humans, alongside the more technically demanding experimental setup required to conduct those experiments, relative to heart rate baroreflex measurements. Ligtenberg and others (Ligtenberg et al., 1999) demonstrated comparable muscle SNA baroreflex sensitivity in hypertensive CKD patients and normotensive controls despite a resetting of the sympathetic baroreflex function to a higher BP range in the CKD group. In contrast, Tinucci and colleagues (Tinucci et al., 2001) demonstrated markedly attenuated muscle SNA baroreflex sensitivity in CKD patients relative to controls; therefore suggesting a pressing need for more clinical studies to elucidate the role of altered sympathetic baroreflex in mediating sympathetic hyperactivity in CKD. As far as animal studies are concerned, a huge body of evidence, as shown by our laboratory work (Harrison et al., 2010; Hildreth et al., 2013; Salman et al., 2014; Salman et al., 2015a; Yao et al., 2015) and others (Chen et al., 2016; Freitas et al., 2016), has consistently supported the presence of impaired cardiac and sympathetic baroreflex function in CKD - results which align more with the clinical data previously put forward by Tinucci and colleagues (Tinucci et al., 2001). Interestingly, over the last 2 decades, an overwhelming amount of attention has been directed toward correction of SNS overactivity, baroreflex dysfunction and high BP in the resistant hypertensive population. Such investigations have involved using pacing of the carotid baroreceptors (Figure 2) to increase their activity (also known as baroreflex activation therapy; BAT) (Bhatt et al., 2019) and hence reflexively reduce SNS central discharge (Illig et al., 2006; Heusser et al., 2010; Bisognano et al., 2011). This can be practically performed via baroreflex amplification therapy (electrically by barostimulator or mechanically by stent) and carotid body modulation (Groenland and Spiering, 2020). Several preclinical studies have been conducted on animals for proof of concept (Lohmeier and Iliescu, 2011), some with remarkable BP lowering effects. For example, Lohmeier and colleagues have shown that following a week of continuous baroreflex activation in dogs, a significant and sustained reduction in BP, heart rate, and NE levels, without a compensatory increase in plasma renin activity was observed (Lohmeier et al., 2004). A few clinical studies demonstrated some clinical benefits for BAT in the CKD population, including significant reductions in BP and improvements in kidney function, which could potentially lower CV risks in those patients (Wallbach et al., 2014; Beige et al., 2015; Wallbach et al., 2018). While baroreflex stimulation appears quite promising in treating resistant hypertension in CKD, longitudinal data are still inadequate and further research involving randomized, sham-controlled trials is required to define the long-term efficacy of this treatment approach and determine its ability to lower CV morbidity and mortality in CKD.

Taken together, SNS overactivity is a classical feature of CKD, and its role in the maintenance and progression of vascular disease in CKD is quite complex, given the well-established SNS interaction with vascular and hormonal mechanisms responsible for BP control. Despite a variety of interventions targeting SNS hyperactivity including non-pharmacological, pharmacological, and surgical interventions many patients remain undertreated, suggesting a pressing need for more innovative treatment approaches for sympathetic overactivity in the CKD population.

### 2.3 Renin angiotensin aldosterone system

In physiological terms, RAS has the capacity to regulate arterial pressure and sodium balance (Figure 1). When BP or perfusion falls, or SNA increases, the juxtaglomerular cells release renin which cleaves angiotensinogen, leading to an increase in Ang II levels (Morgado and Neves, 2012). The octapeptide Ang II has powerful direct vasoconstrictor effects, and stimulates the production of aldosterone, a mineralocorticoid which in turn increases renal sodium reabsorption, thus closing the regulatory feedback loop. If blood volume is normal, the increased RAS activity produces an abnormal rise in BP (Morgado and Neves, 2012). And, in patients with mild to moderate renal insufficiency, elevated plasma renin activity and Ang II concentrations correlate well with BP (Sinclair et al., 1987).

In CKD secondary to vascular disease, diabetes or PKD, reports have indicated that in areas of renal injury or ischemia induced by scarring, there is a greater production of local and intra-renal Ang II, which then exacerbates systemic hypertension (Acosta, 1982; Rosenberg et al., 1994; Fonseca et al., 2014). This increase in the RAS component is often responsible for at least part of the hypertension that persists after the restoration of normovolemia (Vertes et al., 1969; Preston et al., 1996). The hyperactivation of the RAS leads to elevation of peripheral vascular resistance, resulting in a vicious cycle and decline in cardiac performance, progressing to cardiac remodeling (Makaritsis et al., 2006). This is because Ang II itself is a pro-inflammatory mediator that can activate nuclear factor κ-light-chain-enhancer of activated B cells (NF-kB) (Ruiz-Ortega et al., 2000) and vascular adhesion molecule-1 (VCAM-1) (Rodríguez-Vita et al., 2005). At the kidney, Ang II can promote renal fibroblasts, increase expression of transforming growth factor β1 (TGF- β1), tissue growth factor, and fibronectin and collagen type I, which eventually increases renal fibrosis (Wolf, 1998; Lin et al., 2005). Furthermore, Ang II upregulates plasminogen activator inhibitor-1 and tissue inhibitor of matrix metalloproteinases-1, causing accumulation of extracellular matrix (Abrahamsen et al., 2002). In support of this, it has been shown that the AT_1_ receptor antagonist, losartan, can prevent the development of CKD by maintaining renal blood flow, decreasing inflammation and tissue hypoxia following acute renal injury (Rodríguez-Romo et al., 2016).

The role of RAS is most apparent when considering the effectiveness of angiotensin-converting enzyme inhibitors (ACEi) or AT_1_ receptor antagonists/blockers (ARB) in controlling BP in renal insufficiency (Martinez-Maldonado, 1998), reducing muscle SNA (Ligtenberg et al., 1999; Klein et al., 2003b; Neumann et al., 2007), and providing renoprotection beyond BP control (Adamczak et al., 2002). Numerous studies have documented a key role for RAS effects on both the activity of the SNS and vascular remodeling. Development of vascular hypertrophy, remodeling and/or stiffness causes structurally based vascular amplification, which produces an increased sensitivity of mean arterial pressure (MAP) to vasoactive agents, including an augmented response to removal of sympathetic vasoconstriction inputs, which may otherwise appear as an increase in sympathetic activity (Moretti et al., 2009).

Ang II is also known to mediate central sympathoexcitation (Veerasingham and Raizada, 2003; Fink, 2009; Sun et al., 2009). The mechanism by which Ang II is believed to alter sympathetic outflow to organs like the heart and vasculature involves activation of nicotinamide adenine dinucleotide (NADPH) oxidase and increased levels of reactive oxygen species (ROS) in neuroanatomical regions within the brain central to sympathetic stimulation (Gao et al., 2005; Gao et al., 2008; Schlaich et al., 2009). In experimental animal models, renally-mediated local release of Ang II appears to mediate central activation of the SNS activity, as ARBs abrogate SNS activation caused by renal injury in rat experiments (Ye et al., 2002). A study conducted on our CKD rat as alluded to earlier, the LPK model, revealed that acute blockade of AT_1_ receptor by IV losartan ameliorated not only BP but also improved baroreflex control of splanchnic SNA and further reduced the splanchnic, renal and lumbar SNA chemoreflex outflows in this CKD model (Yao et al., 2015); findings which thus support the role of Ang II in perpetuating the peripheral SNS reflexes during CKD progression.

With respect to the effect of Ang II on vascular remodeling in kidney disease, we have conducted a series of different experiments on our CKD rat model that evidently highlights the significance of RAS antagonism in CKD-presented vascular pathology. As an earlier study from our group has demonstrated, chronic administration of the ACEi, perindopril to the LPK rat causes a reduction in the PWV index of arterial stiffness and enhances central artery compliance (Ng et al., 2011). These results were not achieved by renal denervation of our animals (Yao et al., 2017), and whether this is the case in different CKD animal models or humans with kidney diseases has not been fully investigated. Hence, further research in this area is required in order to establish a clear ground on the clinical outcome of RAS antagonism versus that of renal denervation.

Regarding RAS pharmacological antagonism in CKD, the examination of the effect of chronic AT_1_ blockade with valsartan on the function and structure of pathological vascular remodeling were assessed further in the LPK model of CKD in two different studies. In an earlier study, the aorta was used to investigate large artery dysfunction derived by CKD (Ameer et al., 2016). This study revealed that valsartan ameliorated the NE sympathetic mediator constricting effect on the aorta, normalized endothelium-dependent and -independent relaxations, improved NO functionality, and reduced large artery hypertrophy, wall elastin component fragmentation, calcification, vessel cystic degeneration and aortic wall matrix metalloproteinase-9 levels (Ameer et al., 2016). This myriad of effects suggests that AT_1_ antagonism can extend to have effects that are beneficial for CV health. In a later complementary study, the mesenteric artery was utilized to investigate small resistance artery remodeling in response to valsartan (Quek et al., 2018). Here, the chronic AT_1_ antagonism had a detrimental effect on small artery geometry as it increased internal and external diameters and reduced the thickness of mesenteric walls alongside artery wall–lumen ratios. These effects would result in a significant impact on peripheral vascular resistance and vascular amplification (Adams et al., 1990; Smid et al., 1995; Ameer et al., 2014a), of which constitutes a hallmark of peripheral vascular resistance and a high BP that is often difficult to lower. The functional improvements in the large conduit artery seen in the earlier aorta study were also carried out in a relatively similar manner in smaller resistant mesenteric artery, especially with respect to improvement of endothelium dysfunction and NO contribution to relaxation. Furthermore, elevation of endothelium-derived hyperpolarization factor (EDHF) and the downplaying of prostanoids roles in resistant mesenteric artery of this CKD model were found to be a significant component of vascular-modulating autacoids. Interestingly, mesenteric artery biomechanical properties were further improved with valsartan treatment, as resistant artery compliance increased while intrinsic stiffness reduced; such effects could be reflected by changes in artery wall structural configuration of elastin and collagen components (Quek et al., 2018). No doubt, results from these investigations highlight the role of Ang II in driving vascular manifestations in CKD (Quek et al., 2018). Such findings also justify why early treatment with specific pharmacotherapy such as Ang II antagonism can potentially pan effects that not only induce BP reduction in CKD, but could limit pathological changes in arteries and improve the CV health beyond BP control; results of which may not be fully seen with other pharmacological interventions (Jeewandara et al., 2015; Quek et al., 2020). This likely explains why the ACEi and ARB are evidently taking the lead in treatments of hypertensive kidney disease (Unger et al., 2020).

As a result of RAS activation, the hormone aldosterone is produced. In addition to its renal sodium reabsorption effects, aldosterone is involved in various biological processes at the level of the vasculature. For instance, it can induce endothelial dysfunction through various mechanisms including reduction of NO bioavailability, increased inflammation, oxidative stress, and direct effects on glucose-6-dehydrogenase and epidermal growth factor, resulting in increased endothelial cell volume, stiffness and inability to release NO (Briet and Schiffrin, 2013). Aldosterone can also act on endothelial progenitor cells reducing vascular homing, migration, differentiation, and proliferation; the effect of which could cause reductions in circulatory colony-forming units of the endothelial cells and impairment of their migratory function (Briet and Schiffrin, 2013). Studies demonstrating the deleterious aldosterone effects suggest the involvement of this mineralocorticoid in tissue remodeling and fibrosis, as verified in various animal models of hypertension and kidney failure (Greene et al., 1996; Blasi et al., 2003; Nishiyama et al., 2004). Interestingly, increased oxidative stress and vascular inflammation are thought to be the first damaging effects of aldosterone on the vasculature, as it has been shown that in experimental uni-nephrectomized rats receiving aldosterone, a vascular increase in oxidative stress and inflammatory mediators preceded arterial fibrosis (Sun et al., 2002). Our research team has also investigated the effects of chronic aldosterone antagonism with spironolactone on the CV and renal function of the LPK model of CKD (Jeewandara et al., 2015). Unexpectedly, we found that spironolactone reduced BP in female rats only, and not in male rats, exhibiting sexual dimorphism for being resistant to BP reduction in male CKD rats. Furthermore, cardiac perivascular fibrosis, α-cardiac actin marker, and kidney function were evidently reduced with this treatment; however, spironolactone did not rectify the vascular remodeling (Jeewandara et al., 2015) and oversensitivity to constriction was seen in this model; changes that were evidently ameliorated with RAS antagonism as discussed earlier (Ameer et al., 2016; Quek et al., 2018). Thus, we know that aldosterone can exert damaging effects on the kidney and heart, that can have pathophysiological consequences (Hian et al., 2016).

Spironolactone has also been shown to help prevent cardiac and vascular fibrosis independently from reductions in BP (Benetos et al., 1997; Lacolley et al., 2001). For example, administration of spironolactone was found to prevent cardiac hypertrophic remodeling and increase expression of NADPH oxidase components in uremic rats (Michea et al., 2008), which further suggests that oxidative stress, proinflammatory and profibrotic actions of aldosterone are highly relevant pathophysiological conditions by which this mineralocorticoid is implicated in mediating CV injury (Briet and Schiffrin, 2010). A combination of RAAS axis antagonism (i.e. ACEi/or ARB plus aldosterone antagonist) is often used and can have beneficial effects in lowering proteinuria, and resistant BP in CKD patients (Unger et al., 2020); however, the establishment of ESRD progression with this combination still lacks characterization (Bolignano et al., 2014; Chung et al., 2020). Furthermore, this combination increases the risk of hyperkalemia and gynecomastia secondary to aldosterone antagonism, especially with old mineralocorticoid antagonists, and patients with such pharmacological interventions should be carefully monitored. Nevertheless, the development of new non-steroidal mineralocorticoid aldosterone receptor antagonists like finerenone has proven to have a superior safety profile to the old spironolactone and slightly newer eplerenone in heart and kidney disease patients (Capelli et al., 2020; Volpe and Patrono, 2020). The important clinical outcomes of these newly approved drugs on mortality rate, CV events, proteinuria and renal death reduction are yet to be evaluated.

As shown above, it is evident that most of our knowledge on the role of Ang II in CV control is heavily weighed on the understanding of its modulatory action on AT_1_ receptors. Despite decades long of physiological evidence (Johnson and Malvin, 1977; van der Mark and Kline, 1994) describing the counterregulatory role for AT_2_ receptors, which often oppose the AT_1_ action mediated by Ang II, their pharmacological targeting has been far less emphasized in the literature, perhaps due to lower AT_2_ receptor expression levels in adults relative to that of AT_1_ receptors (Ozono et al., 1997). Indeed, animal studies, using models of hypertension, heart and kidney disease and obesity, have shown that AT_2_ receptor activation promotes diuresis and natriuresis, lowers BP, inhibits vascular calcification, suppresses chronic inflammation, mitigates cardiac damage, and improves renal structural and functional abnormalities (Johnson and Malvin, 1977; van der Mark and Kline, 1994; Gelosa et al., 2009; Kukida et al., 2019; Wang et al., 2020; Gavini et al., 2021). Further relating to this is the additive beneficial action for AT_2_ receptor stimulation when AT_1_ receptors are blocked, facilitating preferential stimulation of the unblocked AT_2_ receptors (Kemp et al., 2012). It is therefore due to these beneficial outcomes that this pathway is now commonly referred to as the “*protective arm*” of RAS. A number of AT_2_ receptor agonists have emerged from animal studies including Compound 21 (Kaschina et al., 2008), MOR107 (Wagenaar et al., 2013) and NP-6A4 (Gavini et al., 2021); however, to date, therapeutic effects of these compounds in ameliorating CV and renal diseases have yet to be thoroughly tested in phase II/III human trials. If proven effective, pharmacological targeting of AT_2_ receptors may serve as a potential strategy to treat CV deficits associated with hypertension and CKD.

Collectively, one can acknowledge that RAAS plays a significant role in CKD development and progression, given its multifaceted contribution to the modulation of neuronal, vascular, and inflammatory pathways impacting CV and renovascular control. The efficacy ACEi and AT_1_ receptor blockers and their use as first-line agents to treat CKD patients is not surprising owing to their beneficial actions that extend beyond BP control. Moreover, the emergence of more research evidence regarding the protective role of AT_2_ receptors and their ability to augment the action of ARBs may represent a novel therapeutic approach to treating CV complications associated with CKD.

### 2.4 Role of the vasculature in hypertension in chronic kidney disease

**FIGURE 3:**
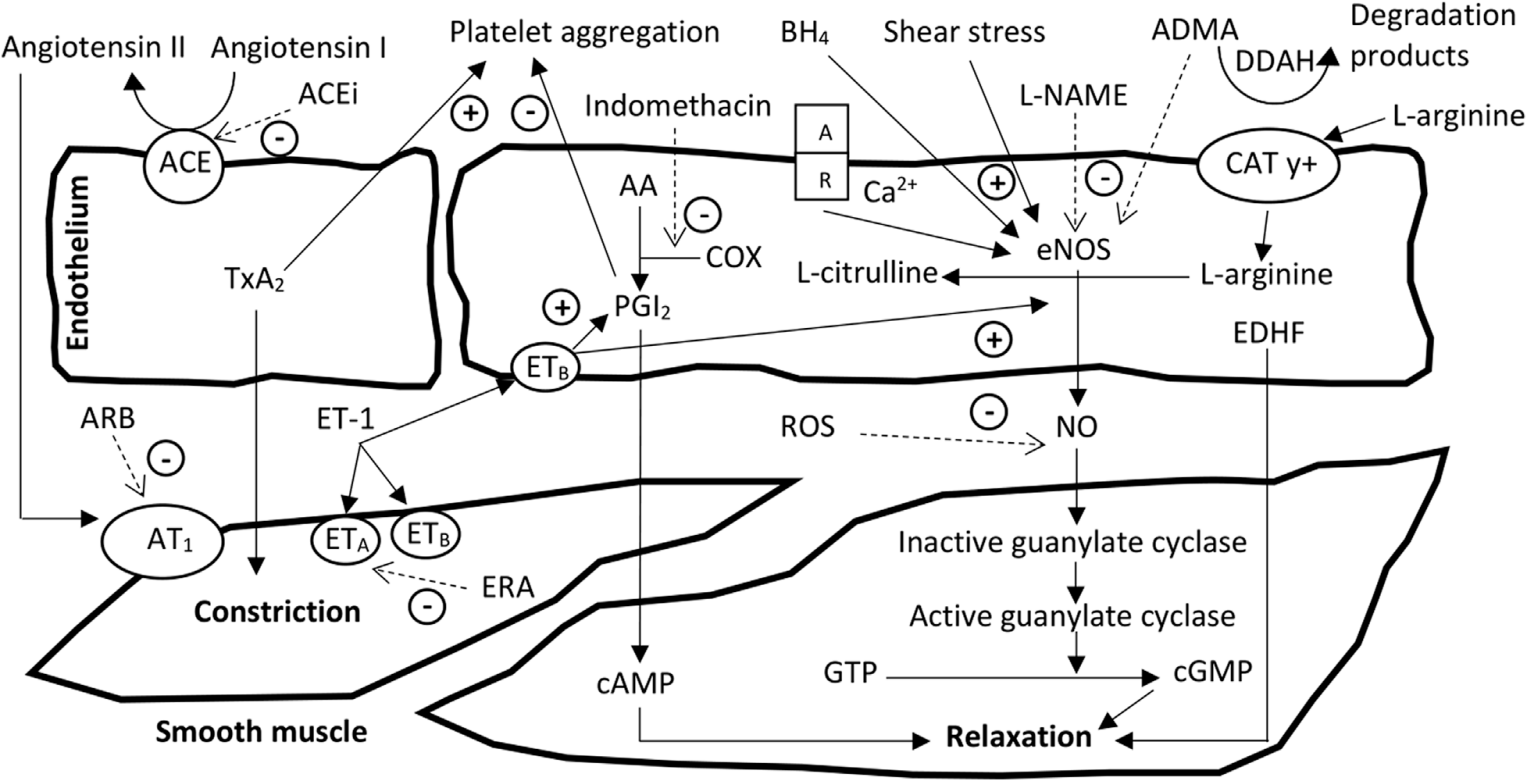
Major signaling pathways involved in endothelial and vascular smooth muscle function. Cationic amino acid transporter-y + regulates uptake of l-arginine substrate into the endothelial cell. Agent A, acting on receptor (R) of the endothelial cell activates Ca^2+^ influx: for example, acetylcholine (ACh) acts on the endothelial muscarinic (M_3_) receptor causing Ca^2+^ influx. The increase in intracellular Ca^2+^ leads to activation of endothelial nitric oxide synthase (eNOS). Shear stress or substrate supplementation of tetrahydrobiopterin (BH_4_) can increase activation of eNOS while the latter is inhibited by asymmetric dimethylarginine (ADMA). Nitric oxide (NO) endothelial derived relaxing factor (EDRF) then diffuses to the smooth muscle cells where it activates guanylate cyclase, which results in an increase in cyclic guanosine monophosphate (cGMP), and thus initiates processes leading to relaxation. N^ω^-nitro-l-arginine methyl ester (l-NAME) is a NOS inhibitor, and reactive oxygen species (ROS) act as NO scavengers. Endothelin is an endothelium-derived peptide, which causes vascular contraction via smooth muscle endothelin A and B receptors (ET_A_ and ET_B_) or relaxation via endothelial ET_B_. Arachidonic acid (AA) is a membrane phospholipid that gives rise to prostanoids via cyclooxygenase (COX). Prostacyclin I_2_ (PGI_2_) and thromboxane A_2_ (TxA_2_) are prostanoids. The former causes vascular relaxation and inhibits platelet aggregation, while the latter produces the opposite of these effects. COX can be inhibited by nonsteroidal anti-inflammatory drugs like indomethacin and thus inhibit prostanoid formation. Angiotensin converting enzyme (ACE) produces angiotensin II (Ang II); Ang II can cause smooth muscle contraction via angiotensin-1 receptor (AT_1_) activation. ACE inhibitor (ACEi) blocks Ang II formation. Angiotensin receptor blockers (ARB) block angiotensin action on the AT_1_ receptor and endothelin receptor antagonists (ERA) inhibit endothelin action on the ET receptors. Adapted and modified from (Cohen and Vanhoutte, 1995; Félétou and Vanhoutte, 2006; Wong and Vanhoutte, 2010; Raina et al., 2020; Roumeliotis et al., 2020).

One issue contributing to the high BP state in CKD is the presence of vascular changes. The vessels of CKD patients are continuously exposed to numerous hormones, factors and uremic toxins that can be responsible for the observed vascular changes and dysfunction, which in turn causes a rise in BP that is often difficult and resistant to treatment with drug therapy. Vascular dysfunction can take place early in the progression of CKD and although it appears to be independent of hypertension development, it is considered a definite part in the hypertension sequalae (Schiffrin et al., 2007; Ameer et al., 2015; Six et al., 2020). As mentioned earlier with our findings from a CKD animal model, vascular changes may be structural, as characterized by vessel remodeling that favors increased contractility and a loss in elasticity causing vascular resistance (Ameer et al., 2014b; Ameer et al., 2016; Quek et al., 2016); or they may be associated with functional changes such as the regularly encountered defect in acetylcholine (ACh) relaxation (Figure 3) (Morris et al., 2000; Ameer et al., 2015; Roumeliotis et al., 2020; Six et al., 2020). Such changes are often associated with an overexpression of the intracellular adhesion molecule-1 (ICAM-1) and VCAM-1 (Safar et al., 2003; Rodríguez-Vita et al., 2005; Six et al., 2020). We know thus far these vascular alterations have been associated with the development of cardiac pathologies, further raising BP and worsening during CKD progression (Maizel et al., 2009). Of note is that the consistent observation of endothelium dysfunction in CKD patients can be the result of an increase in endothelial injury and a reduction in endothelial repair (Goligorsky et al., 2010), and this dysfunction has been associated with numerous systemic BP and hemodynamic alterations.

#### 2.4.1 Endothelial dysfunction

Endothelial dysfunction can be described as a systemic pathological state of the endothelium (the inner most lining of blood vessels) that arises from an imbalance between vasodilating and vasoconstricting substances produced by (or acting on) the endothelium (Deanfield et al., 2005). This is because the endothelium itself acts as a dynamic and functionally complex organ that is involved in the regulation of many crucial biological mechanisms, including maintenance of vascular tone and permeability, inflammatory responses, immunity, and angiogenesis (Roumeliotis et al., 2020).

In patients with CKD, endothelial dysfunction may have a dual role, for not only is it a crucial step in the development of CVD, but dysfunction and disintegration of endothelial cells in glomerular capillaries, and particularly those capillaries in renal medulla pave the way for CKD progression (Fliser et al., 2011; Harlacher et al., 2022). Furthermore, the endothelial dysfunction may serve as an early basis of atherosclerosis during the early stages of CKD, and the prevalence of this dysfunction is progressively high toward later ESRD (Yilmaz et al., 2006). Importantly, the loss of endothelium integrity is highly paralleled by increased mortality rates from CVD incidence (Roumeliotis et al., 2020).

There are many endothelium-derived relaxing factors (EDRFs) and endothelium-derived constricting factors (EDCFs) that have been speculated to play a role in hypertension associated with kidney disease (Shultz, 1992; Martinez-Maldonado, 1998; Zhao et al., 2021). These and pharmacological agents that are commonly used to target or influence them, are shown in Figure 3.

Endothelium dysfunction can have an impact on the function and structure of the vasculature. Functionally, impaired endothelium-dependent vasodilation has been observed in CKD and ESRD patients (Passauer et al., 2000; Thambyrajah et al., 2000; Wang et al., 2000; Amabile et al., 2005), and has been described as a pervasive phenomenon in uremic individuals, which may later influence morbidity and mortality from large vessel atherosclerotic disease and LVH (Zoccali et al., 2003). Microalbuminuria, a marker of glomerular hyperfiltration, has been correlated with and may be a manifestation of impaired endothelial function (Stehouwer and Smulders, 2006), and is suggested to participate in the mechanisms that lead to progression of renal disease (Fujihara et al., 1995). Indeed, it has been postulated that glomerular endothelial dysfunction is an early feature of essential hypertension that may precede BP elevation (Schiffrin et al., 2007). Structurally, endothelium dysfunction is implicated in arterial remodeling by several mechanisms, including inhibition of platelet aggregation and monocyte adhesion to the endothelial cell, abrogation of low-density lipoprotein (LDL) cholesterol oxidation, and atherosclerosis (Pannier et al., 2000; Van Varik et al., 2012; Roumeliotis et al., 2020). Furthermore, endothelium influences vascular wall homeostasis via the endothelial cell cytokines and growth factors (Csiszar et al., 2009; Urschel et al., 2012; Van Varik et al., 2012). Endothelial cells can also affect the state of vascular hypertrophy via the production of TGF-β and bone morphogenetic proteins (BMPs) which stimulate vascular smooth muscles and pericytes to remodel and deposit extracellular matrix (Boström et al., 1993; Simionescu et al., 2005; Van Varik et al., 2012). Therefore, the preservation of the functional and structural integrity of the vascular endothelial cells is fundamental for not only vasculature hemodynamics, but also the global CV health state.

#### 2.4.2 Nitric oxide signaling

Vascular endothelial cells cause vasodilatation as a normal response to increases in flow, shear stress and agonists through the release of several mediators, which include NO, prostaglandin I_2_ (PGI_2_) and EDHF (Figure 3) (Campese et al., 2006; Roumeliotis et al., 2020).

Nitric oxide is a gaseous molecule with vasorelaxant, anti-inflammatory, and antithrombotic properties (Vanhoutte et al., 2016), and is probably one of the most important molecules produced by the endothelium. Reduced NO bioavailability (Figure 1) appears to be one of the main factors involved in CKD-associated endothelial dysfunction and its hallmark (Wever et al., 1999; Hasdan et al., 2002; Passauer et al., 2005; Roumeliotis et al., 2020).

Nitric oxide is synthesized by the enzyme nitric oxide synthase (NOS) from the precursor amino acid l-arginine (L-Arg) (Figure 3). Nitric oxide synthase is present in three isoforms named after the different tissues they were initially discovered in (Lüscher and Vanhoutte, 1990; Bruno and Taddei, 2011), though each distinct isoform has since been discovered to also be expressed in other tissues. Neuronal NOS (nNOS) is constitutively expressed in central and peripheral nervous system neurons, where NO acts as an important neurotransmitter and plays a key role in the autonomic regulation of CV function (Patel et al., 2001; Hirooka et al., 2011), and is also expressed in skeletal muscle and the epithelial layers of the airways. Neuronal NOS is also present in macrophages and endothelial cells, where it seems to play a role in the regulation of basal vascular tone (Seddon et al., 2008), and has been implicated in the rise of central SNS activity in CKD patients (Park et al., 2015). Inducible NOS (iNOS) is expressed in a number of different cellular types and its activity is minimal under baseline conditions, but it is progressively induced by inflammatory stimuli (Lüscher and Vanhoutte, 1990) and was originally discovered in macrophages. Endothelial NOS (eNOS) is constitutively expressed not only in the vascular endothelial cells, but in various cell types including neuronal cells (Bruno and Taddei, 2011), myocardial cells, and skeletal muscle. Nitric oxide released from the endothelium is triggered by receptor-mediated mechanisms including ACh, bradykinin, serotonin, substance P, adenosine diphosphate and triphosphate, histamine and endothelin-1 via endothelin B receptors, which are all Ca^2+^-dependent (Figure 3) (Roumeliotis et al., 2020); and by nonspecific mechanical stimuli such as shear stress by blood flow or hormonal growth factors like vascular endothelial growth factor, insulin and adiponectin - all of which are Ca^2+^-independent mechanisms (H Heiss and M Dirsch, 2014; Roumeliotis et al., 2020). Remarkably, the shear stress on the vascular walls is the most powerful mechanism of stimulated NO release (Bruno and Taddei, 2011). Endothelial-derived NO has the capacity to maintain vascular tone and to produce vasodilatation through the activation of guanylate cyclase and cyclic guanosine monophosphate (cGMP) (Figure 3) (Bruno and Taddei, 2011; Roumeliotis et al., 2020).

It has been reported that various factors can impair the NO pathway during CKD and consequently cause further elevation in BP (Figure 1). The oxidative stress state is one example, whereby an imbalance between antioxidants and oxidants leads to potential damage (Sies, 1997; Vaziri et al., 1998; Daenen et al., 2019). Thus, reductions in antioxidant mechanisms such as catalase, superoxide dismutase (SOD) and glutathione dismutase (Vaziri et al., 2002); and elevation in oxidation mechanisms such as superoxide, hydrogen peroxide (Vaziri et al., 2002), activation of the reduced form of NADPH oxidase, xanthine oxidase, uncoupled eNOS, myeloperoxidase, and mitochondrial oxidases (Vaziri et al., 2002; Daenen et al., 2019) could be potential pathways that lead to an oxidative stress state, which would result in increased ROS in uremia. Uncoupling of eNOS under pathological conditions results in ROS production, caused by electron transfer within eNOS active sites to uncouple it from L-Arg and oxygen reduction to superoxide free radical (O_2_^•-^) (Alp and Channon, 2004).

In addition, it has been demonstrated that vascular smooth muscle NADPH oxidase is activated by Ang II and subsequently increases vascular ROS levels (Griendling et al., 1994), with the NADPH p47 (phox) subunit being probably one of the most important oxidation sources in the vasculature that is also upregulated by Ang II (Touyz et al., 2005); a finding which has been evident with the increased aortic expression of p47 phox toward ESRD in a CKD rat model (Ameer et al., 2015). The oxidant free radical rapidly reacts with NO to form the highly reactive intermediate peroxynitrite (ONOO^−^) (Daenen et al., 2019). The formation of these nitroso-compounds has multiple negative effects, reducing NO availability, exerting direct vasoconstriction, and impairing the activity of prostacyclin synthase and eNOS (Munzel et al., 2010), thus contributing to an increase in BP in CKD (Hu et al., 1998). Furthermore, it has been established that lipid oxidation products such as oxidized LDL promote the displacement of eNOS from its surface membrane caveolae to the cell cytoplasm, where its activity is consequently directly inhibited (Blair et al., 1999; Gharavi et al., 2006; Roumeliotis et al., 2020). This occurrence can be translated to the inverse relationship between oxidized-LDL with vascular distensibility, such as the carotid artery in CKD patients (Nawrot et al., 2010).

Renal failure also plays a role as a trigger for inflammatory responses, which influence NO bioavailability (Schiffrin et al., 2007). Hypoxia, tumor necrosis factor-α and inflammatory cytokines are main stimuli that can negatively influence eNOS expression (Lüscher and Vanhoutte, 1990). Moreover, accumulation of inflammatory markers such as C-reactive proteins, uremic toxins, advanced glycation end products (AGE) (Vaziri et al., 1998; Jofré et al., 2006) and Ang II-mediated amplification of inflammatory mechanisms (Pecoits-Filho et al., 2003) can also impact NO bioavailability negatively. It has been demonstrated that cell transfusion of healthy endothelial cells into 5/6 nephrectomized rats improves NO-mediated endothelial dysfunction in the renal failure state (Pacurari et al., 2013). Therefore, it is likely that the concomitant uremia state associated with renal impairment can be a trigger for diminished NO bioavailability and progressive endothelial dysfunction; a vascular calamity that can further deteriorate renal function in CKD (Schiffrin et al., 2007; Roumeliotis et al., 2020).

The L-Arg and pteridine cofactor tetrahydrobiopterin (BH_4_) are essential precursors for NO production (Figure 3) (Daenen et al., 2019), and their reduction has been associated with limiting NO bioavailability and decreased endothelium-dependent relaxation (Moreau et al., 2012; Zhang et al., 2012). Supporting the former precursor involvement, we found that acute addition of L-Arg *in vitro* vascular preparations modestly improves the endothelium-dependent relaxation of the aorta in a polycystic CKD rat model (Ameer et al., 2015; Ameer et al., 2016). Although transiently demonstrated, this effect would roll out the absence of defects in L-Arg uptake through the endothelial cationic transporter (CAT y+) of L-Arg (Figure 3) (Schiffrin et al., 2007; Roumeliotis et al., 2020), at least in the early stages of renal impairment of this CKD model. Despite that there has been no absolute cutoff regarding the benefits or harm of the acute or chronic L-Arg supplementation in CKD (Peters et al., 1999; Cross et al., 2001; Düsing et al., 2021), L-Arg deficiency is becoming a leading factor for eNOS deficits, and its substitution can show some positive CV effects in renal insufficiency (Amador-Martínez et al., 2019; Düsing et al., 2021; Hsu and Tain, 2021). Note however that others have reported that chronic L-Arg intake might predispose to endothelial cells aging (Scalera et al., 2009), and its acute intake does not improve the endothelial dysfunction encountered in kidney disease (Cross et al., 2001). With respect to the effects of BH_4_ on CV health, several studies have demonstrated a role for this NOS re-coupler in various conditions, including but not limited to: improving endothelial function and arterial stiffness in post-menopausal women (Moreau et al., 2012), oxidative stress in arterial diseases (Maier et al., 2000; Cosentino et al., 2008), hypertension (Porkert et al., 2008), NO-mediated vascular relaxation in type II diabetes (Heitzer et al., 2000), protection against atherosclerosis and vascular inflammation in a mice model (Schmidt et al., 2010), reduced elevated BP, improved kidney function in 5/6 nephrectomy rat model (Podjarny et al., 2004), reduced arterial wall thickness and collagen remodeling in a chronic ischemia rat model (Akaihata et al., 2020), and reversal or alleviation of vascular pathologies associated with CKD (Ameer et al., 2014b; Ameer et al., 2015; Quek et al., 2016). Despite the aforementioned, it is still critical to know that certain underlying etiologies like oxidative stress may still hamper the beneficial effects of BH_4_ treatment, especially in kidney disease (Channon, 2020). It is therefore crucial to revisit the effect of these bio-precursors and reveal their key role in NOS-mediated activities, especially in the treatment of the high BP state in CKD animal models and humans, and research and optimization of NO re-coupler and precursors are of importance within this area of NO-linked vascular pathology.

Another feature associated with impaired NO signaling is the accumulation of asymmetric dimethylarginine (ADMA) (Figures 1, 3) (Channon, 2020), and its less researched inactive enantiomer symmetric dimethylarginine (SDMA) (El-Khoury et al., 2016; Six et al., 2020). Asymmetric dimethylarginine, a naturally occurring amino acid, is an endogenous competitive inhibitor of NOS (Vallance and Leiper, 2004), which can cause endothelial dysfunction and/or reduced NO production particularly in renal failure (Leone et al., 1992; Zoccali et al., 2001), and is associated with increased CV risk (Bruno and Taddei, 2011). It is also reported to be elevated in CKD (Leone et al., 1992; Fleck et al., 2001) and ESRD patients on dialysis (Anderstam et al., 1997). Interestingly, ADMA levels have been found to correlate with the severity of atherosclerosis (Kielstein et al., 1999), CV events, and mortality in CKD patients (Zoccali et al., 2001), and even with renal disease state progression (Fliser et al., 2005). Asymmetric dimethylarginine levels can be influenced by Ang II through the activation of AT_1_ receptors; thus, Ang II can indirectly predispose to further NOS inhibition (Palm et al., 2007), perpetuating to eNOS uncoupling with free radicals generation (Vallance and Leiper, 2004). Asymmetric dimethylarginine is mainly metabolized by dimethylarginine dimethylaminohydrolase (DDAH) and cleared by the kidney (Figure 3) (Schiffrin et al., 2007; Roumeliotis et al., 2020).

The synthesis of ADMA requires the enzyme protein arginine methyltransferase, which methylates arginine residues (Schiffrin et al., 2007; Channon, 2020). Asymmetric dimethylarginine enters vascular endothelial cells via the same CAT y + transporter of L-Arg (Figure 3). The activity of this transporter colocalizes with caveolin-bound NOS, which suggests that CAT y + transporter activity may be a determinant of the local concentrations of ADMA. The ADMA then competes with L-Arg substrates for transport into the cell. Thus, ADMA may block entry of L-Arg, resulting in a decrease in NO synthesis (Schiffrin et al., 2007; Channon, 2020). Adding to these complexities, DDAH-mediated catabolism of ADMA is severely hampered by the presence of circulating inflammatory cytokines, ROS, hyperlipidemia, hyperglycemia and their related consequence including AGEs (Roumeliotis et al., 2020); many of which are associated with the uremic state in CKD (Schiffrin et al., 2007; Roumeliotis et al., 2020).

#### 2.4.3 Prostanoids signaling

The prostanoids are eicosanoids derived from autocoids from the membrane of the cell, and are part of the EDRFs and EDCFs (Zhao et al., 2021). The prostanoids are a family of lipid mediators generated by the action of cyclooxygenase (COX) on a 20-carbon unsaturated fatty acid, arachidonic acid. Two major isozymes encoded by different genes, COX_1_ and COX_2_, mediate this production process (Smith et al., 2000) and are differentially effective, mainly as vasodilators and vasoconstrictors regulating vascular tone (Figure 3) (Qi et al., 2002; Zewde and Mattson, 2004), while the newly discovered variant COX_3_ enzyme is still under investigation with particular importance in brain tumors (Zhao et al., 2021). Prostanoids encompass prostaglandins (PGI_2_, PGD_2_, PGE_2_ and PGF_2_α) and thromboxane (TxA_2_), which are generated in a paracrine or autocrine manner, and are all implicated in CVD (Smyth et al., 2009; Wu et al., 2021), especially in conditions associated with a state of downregulation of EDRFs and upregulation of EDCFs, such as hypertension (Liang et al., 2019). Prostanoid analogues are becoming a good therapeutic key in the treatment of pulmonary hypertension (Pan et al., 2020) while their synthesis inhibitors like the NSAIDs are often associated with CV and renal deleterious effects, in particular in polypharmacy-treated patients (Cabassi et al., 2020). Prostaglandin I_2_ is most abundant in the aorta followed by TxA_2_, PGE_2_, and PGF_2_α (Qi et al., 2006). Because of their short half-life, prostanoids neither circulate nor impact directly on systemic vascular tone. However, they may modulate local vascular tone at the site of their formation and affect systemic BP (Smyth et al., 2009; Wu et al., 2021). Prostanoids are involved in maintaining normal homeostasis and kidney function, thus PGI_2_ deficiency can induce renal fibrosis and vascular injury, including arteriosclerosis and wall thickening of the aorta. Prostaglandin I_2_ signaling is essential to oppose vasoconstrictor pathways for maintenance of renal homeostasis, and is an important vasodilatory renal prostanoid, which can potentially influence numerous pathological mechanisms implicated in various aspects of renal disease, including renal hemodynamic changes, changes in GFR, oxidative stress, inflammatory processes, and platelet aggregation in renovascular disease complicated by hypertension (Nasrallah and Hébert, 2005; Zhao et al., 2021). While, the downstream of COX_2_ derived prostanoids like TxA_2_ is a key contributing factor in mediating vasoconstriction (Figure 3) and is implicated in endothelial dysfunction and raising BP (Zhao et al., 2021). Furthermore, it has been suggested that impairment of prostanoids-mediated relaxation may contribute to endothelial dysfunction in the aorta of hypertensive rats (Tang et al., 2008). Experimental evidence revealed that upon chronic Ang II inhibition by ACEi, suppression of vasoconstrictor prostanoids production and improvement of flow-mediated dilation in arteries from congestive heart failure rats were achieved (Varin et al., 2000). It is noteworthy to mention that oxidative stress causes an upregulation of COXs in hypertension (Paravicini and Touyz, 2008) and CKD, accelerating CVD incidence (Mathew et al., 2017), and the high uremic solute indole-3 acetic acid triggers COX_2_ expression elevation through endothelial inflammation and oxidation (Dou et al., 2015). Thus, the existence of positive feedback between ROS and COX, and vasoconstricting prostanoids altogether exacerbate BP elevation. Furthermore, a crosstalk relationship between Ang II and the COX system exists, whereby COX activity has been shown to modulate the pressor effect of Ang II on BP (Qi et al., 2002), and Ang II itself acts as a COX_2_ inducer (Zhao et al., 2021). With current advances in research, there is mounting evidence suggesting that the therapeutic potential of drug discoveries is partially dependent on their ability to target prostanoid biosynthesis and their receptors in the treatment of various CVDs including hypertension and nephropathy. Thus far, research efforts are still required to investigate and pinpoint the exact contribution of targeting COX or particular prostanoid receptors, and to clarify the misconception arising from animal research and human studies relating to prostanoids as a therapeutic target.

#### 2.4.4 Endothelium-derived hyperpolarizing factor

Although EDHF’s exact identity remains unknown, EDHF is evidently an important NO, PGI_2_-independent agonist induced relaxation pathway (Figure 3) (Luksha et al., 2009), and TxA_2_ receptor antagonist (Zhao et al., 2021). It acts via relaxing the vascular smooth muscle cell (VSMC) through putative potassium channels and desensitizes the cell to vasoconstrictor stimuli (Chen and Suzuki, 1990). Notably, reserved EDHF protection may serve as a compensatory vasodilatory mechanism in the disease state (Katz and Krum, 2001; Quek et al., 2016; Lobov and Ivanova, 2020). Reports have demonstrated the presence of extensive interaction between NO and EDHF, and that EDHF activity might be enhanced or depressed depending on the state of hypertension, vessel studied, and species (Luksha et al., 2009; Lobov and Ivanova, 2020). For example, EDHF responses were shown to be preserved in mesenteric arteries from the LPK (Quek et al., 2016) and one kidney-one clip Sprague-Dawley rat model of renovascular hypertension (Christensen et al., 2007), yet diminished in the mesenteric arteries from Wistar rats of the same renal failure model (Vettoretti et al., 2006). In contrast, others have reported upregulation of EDHF responses in carotid arteries from two kidney-one clip Wistar rats (Sendao Oliveira and Bendhack, 2004), and an impairment of EDHF mediated relaxation in response to bradykinin but not ACh in subcutaneous resistance arteries from uremic patients (Luksha et al., 2012). Despite this mixed evidence, EDHF as a part of endothelium mechanisms are indeed an important NO deficiency backup component, and are implicated in aggravation of the hypertensive state and end-organ damage (Luksha et al., 2009).

Endothelial dysfunction may result in abnormalities that are not manifested as reduced endothelium-dependent vasodilation (Deng et al., 1995). For example, a dysfunctional endothelium can be associated with changes of endothelial cell phenotypes with increased proliferation, anoikis, altered morphology, production of C-reactive protein, and other inflammatory and thrombogenic mediators, including monocyte chemotactic protein-1 and plasminogen activator inhibitor-1, upregulated adhesion molecules, and enhanced thrombogenicity and adhesiveness for circulating cells (Safar et al., 2003). In a similar fashion reviewed here with respect to oxidative stress affecting NO and prostanoids, ROS and, most importantly superoxide anion, decreases EDHF-induced vasorelaxation; a process which involves decreasing the activity of calcium-sensitive potassium channels and acting on cellular junctions, which thus alters the spread of hyperpolarization of the endothelial cells at the VSMCs (Griffith et al., 2005; Kusama et al., 2005).

#### 2.4.5 Endothelin signaling

Endothelin is a family of four 21 amino acid peptides, and endothelin-1 (ET-1), the main isoform, is produced by endothelial cells. Although the other potent vasoconstrictors and pro-fibrotic growth factors isoforms ET-2 and ET-3 are known (Yanagisawa et al., 1988), ET-1, is the predominant vascular isoform with the greatest regulatory effect on vascular tone, and is integral in the regulation of renal and CV pathophysiology (Raina et al., 2020). Endothelin can mediate vasoconstriction when it binds to its A and B receptors (ET_A_ and ET_B_) in VSMCs, vasodilation ET_B_- mediated via prostacyclin, NO release from the endothelial cells (Figure 3) (de Nucci et al., 1988), and natriuresis in the kidneys (Schneider and Mann, 2014). Nevertheless, endothelin vasoconstricting effect outweighs the vasorelaxing effect, as the latter remains very small and is almost negligible (Channick and Rubin, 2002). It has been found that ET-1 contributes to the pathogenesis and maintenance of hypertension, arterial stiffness and enhancement of CV risk factors such as oxidative stress, inflammation, and endothelial dysfunction (Dhaun et al., 2006; Dhaun et al., 2011; Raina et al., 2020). Research evidence also supported a role for endothelin in regulating autonomic function, demonstrating a modulatory action for endothelin on carotid bodies, superior cervical ganglion and nodose ganglion to influence baroreflex and chemoreflex regulation. Further, a role for endothelin in promoting catecholamine release from the sympathetic nerve terminals and adrenal glands also has been shown (Mortensen, 1999; Salman, 2015). In the kidney, ET-1 via ET_A_ facilitates fibrotic pathways to cause nephropathy progression in conditions such as hypertension, diabetes, dyslipidemia, glomerular sclerosis, and autosomal dominant PKD (Wesson, 2006; Dhaun et al., 2011; Kohan and Barton, 2014; Raina et al., 2020). Furthermore, ET-1 expression may be pathologically enhanced in kidney disease via uremic toxins of AGEs (Six et al., 2020).

A significant crosstalk exists between the RAAS and ET system (Figure 1), as Ang II induces aldosterone release from the adrenal cortex, which in turn increases renal ET-1 expression (Mennuni et al., 2014). Concurrently, renal ET-1 expression is also increased secondary to a rise in vascular NADPH oxidase, NF-kB and ROS due to Ang II actions (Mennuni et al., 2014; Raina et al., 2020). The resultant increased ET-1 activation causes severe renal cortex and medullary vasculature constriction, which eventually reduces renal blood flow and initiates renal fibrosis, a process already paved by the profibrotic effects of ROS and NO level elevations and reductions, respectively (Mennuni et al., 2014).

Endothelin receptor antagonists (ERAs) (Figure 3) have been implicated both in human and animal research studies, especially when needed to tackle hypertension and kidney disease (Goddard et al., 2004; Kohan and Barton, 2014; Breyer and Susztak, 2016; Trachtman et al., 2018; Raina et al., 2020); the ultimate etiologies for ESRD (Mennuni et al., 2014). For example, in the experimental rat model of hypertension and NO dysfunction with deoxycorticosterone acetate (DOCA)-salt and nitric oxide synthase inhibitor Nω-nitro-l-arginine (NOARG), a prototype ET_A_ antagonist treatment produced a major protection against hypertensive nephron damage evidently mediated via NF-kB and ET-1/ET_A_ pathways (Kimura et al., 2012). Interestingly, it has also been demonstrated that the ET_A_ antagonist avosentan, in combination with the ARB antagonist valsartan, gave rise to albuminuria reduction and improved mortality rate in a transgenic hypertensive rat model, relative to the ARB monotherapy (Baltatu et al., 2014); hence supporting the existence of a reno-protective effect for ERA and crosstalk effects of ET with RAS. Such effects have given rise to the clinical study DUET on the new and first in its’ class sparsentan, which acts as a dual ET_A_ and AT_1_ antagonist (Trachtman et al., 2018). In this study, the dual blockade by sparsentan was compared to the conventional ARB irbesartan in focal segmental glomerulosclerosis kidney disease patients, and the results showed that sparsentan was tolerable for up to 8 weeks of treatment with a better reno-protection and proteinuria reduction against that of irbesartan (Trachtman et al., 2018).

Despite the promising research pointing to ERAs as a possible therapeutic option in hypertensive nephropathy, and due to the widespread physiological activity of endothelin across multiple organ systems, ERAs use has unfortunately been accompanied with significant adverse effects. Such effects have ranged from the dangerous and common fluid retention, to exacerbating congestive heart failure, and also included less common hepato-and testicular toxicities and teratogenicity (Kohan and Barton, 2014; Raina et al., 2020). Thus, these side effects severely restrict patients’ eligibility for treatment and limits its clinical usefulness. Current standard available therapy involves ACEi/ARB and may also include the increasingly popular use of sodium-glucose cotransporter-2 inhibitors (SGLT_2_i); hence ERAs are used less often, and when or if considered, involve very patient-specific cases. Certainly, more preclinical and clinical studies are needed to be conducted on certain underlying conditions of hypertension and kidney disease.

### 2.5 Miscellaneous

#### 2.5.1 Parathyroid hormone

Parathyroid hormone (PTH) rises early in CKD and is a culprit contributor to its uremia toxicity. A combination of a decline in kidney function, impairment in phosphate excretion, and failure to synthesize calcitriol the bioactive form of vitamin D, all collectively lead to this maladaptive process of rising PTH level and parathyroid hyperplasia (Cunningham et al., 2011). It has been established that the phosphatonin fibroblast growth factor-23 (FGF-23) has a central role in the regulation of phosphate-vitamin D homeostasis, as this FGF-23 concentration increases secondary to hyperphosphatemia in CKD (Slatopolsky et al., 1999; Six et al., 2020). The progression to secondary hyperparathyroidism causes elevation in the intracellular calcium and plasma phosphate concentrations, and these play their part in increased vascular constriction and raising BP (Raine et al., 1993; Six et al., 2020), where the PTH itself also directly affects vascular tone (Rubin et al., 2005).

Hyperparathyroidism is highly associated with vascular calcification in dialysis patients (Moldovan et al., 2010) and should be considered in risk stratification for kidney-linked mortality in CKD patients (De Nicola et al., 2014). Parathyroidectomy may result in BP improvement in CKD (Morgado and Neves, 2012), and lowering PTH production by the chronic administration of an active vitamin D can reduce both intracellular calcium and systemic BP (Raine et al., 1993). Furthermore, administration of sevelamer as a calcium-free phosphate binder to lower phosphate has also proven to be good for control of serum PTH and FGF-23 levels (Six et al., 2020), and hence would slow the progression of vascular calcification in CKD. Adding to its ability to control phosphate, sevelamer also has pleiotropic effects that can be useful in correcting lipid abnormalities, reducing oxidative stress and inflammatory markers (Nikolov et al., 2006).

#### 2.5.2 Erythropoietin

Erythropoietin, implicated in the treatment of anemia associated with CKD, is overlooked as a causative agent for BP elevation (Krapf and Hulter, 2009; Agarwal, 2018). Most hypertension cases take place with erythropoietin in conditions of severe renal impairment and with dialysis (Krapf and Hulter, 2009), while others of animals or human studies reporting the administration of erythropoietin with normal kidneys showed less advanced BP effects (Agarwal, 2018). Nevertheless, advanced increases in BP associated with erythropoietin use can be triggered by numerous mechanisms, such as: the degree of elevation in the hematocrit or blood viscosity (Raine, 1988; Eschbach et al., 1989), enhanced pressor responsiveness to NE and Ang II, increased cytosolic calcium in VSMCs, increased blood serotonin or endothelin levels (Vaziri, 1999), and increased vasoconstricting (PGF_2α_ and TxA_2_) and decreased vasorelaxing PGI_2_ prostanoid release (Agarwal, 2018). Endothelial dysfunction and the consequent augmented hypertension (Vaziri and Zhou, 2009; Agarwal, 2018) can result in vascular smooth muscle thickening and remodeling, inhibition of endothelial NO through increased production of ADMA (Scalera et al., 2005; Vaziri and Zhou, 2009), increased oxidative stress (Rancourt et al., 2010; Vaziri, 2010), or signaling through ET_A_ receptor (Briet et al., 2013).

#### 2.5.3 Ion channels and signaling

Abnormal signaling pathways through ion channels on the vascular wall may predispose to elevated BP in CKD (Martinez-Maldonado, 1998; Li and Fung, 2019). For example, transient receptor potential cation channels linked to ion transport are also reported to play a role in the pathogenesis of arterial systemic hypertension in CKD (Mene et al., 2013), through regulation of vascular smooth muscle contraction, renal perfusion or hemodynamics, as well as the total body balance of divalent cations. Experimental evidence suggests that expression and/or activity of the depolarizing voltage-gated calcium channels is enhanced during hypertension, whereas the function of the hyperpolarizing potassium channels may be declined (Martinez-Maldonado, 1998). A direct link exists between the density of epithelial sodium channels (ENaC) and vascular functionality. Indeed, overexpression of ENaC promotes arterial stiffness, increased myogenic tone, and endothelium morphology dysregulation and remodeling (Li and Fung, 2019), potentially contributing to heightened BP. Increased activity of protein kinase and increased cytosolic calcium levels may further inhibit the activation of potassium channels, thus increasing the likelihood of arterial smooth muscle depolarization, raising vascular tone, and ultimately arterial pressure (Sobey, 2001).

Interestingly, the effect of endogenous digitalis-like substances or more accurately its close relative ouabains on sodium or volume-dependent hypertension was reported as an associated factor in driving the high BP state in CKD (Morgado and Neves, 2012; Blaustein and Hamlyn, 2020). These include quite a few substances produced in the adrenal gland or in the hypothalamus that additionally cause inhibition of Na-K-ATPase in the cell membranes, and are natriuretic and vasoconstrictors (Takahashi et al., 2011). The elevated levels of these digitalis-like substances in CKD (Hamlyn et al., 1996; Komiyama et al., 2005; Blaustein and Hamlyn, 2020), secondary to an increased sodium content and hypervolemia, may also contribute to the hypertension commonly seen in CKD patients.

Lastly, it is progressively important to know the beneficial effects of SGLT_2_-inhibition with drugs like canagliflozin or empagliflozin is widely focused on CV volume and glycemic control via the induction of natriuresis and osmotic diuresis (Lytvyn et al., 2017). However, the success of SGLT_2_i in slowing CKD progression with and without diabetes (Wanner et al., 2016; Heerspink et al., 2020), may be related to their bonus effects on reducing oxidative stress, vascular alterations and providing significant renoprotection (Fernandez-Fernandez et al., 2020).

#### 2.5.4 Uremic state

In the past 2 decades, investigations using uremic serum from CKD patients have been unravelling the complex composition of uremic toxins and their deleterious effect on BP and kidney disease progression. Those uremic toxins can affect endothelial cell function and viability, via inducing endothelial cell apoptosis, and mitigating its proliferation and migration. For example, following cell activation or apoptosis, the cellular plasma membrane shed what is known as the endothelial microparticles/fragments, which are remarkably increased in CKD (Al-Massarani et al., 2008). These microparticles act to express externalized phosphatidylserine group and endothelium specific antigens, hence culminating into vascular dysfunction (Amabile et al., 2005), impairment of NO signaling pathway, and hypertension in the long run. Other uremic toxins like inorganic phosphate, p-cresyl sulfate, and indoxyl sulfate are known toxins to increase endothelial microparticles shedding and apoptosis, causing augmentation of vascular contractility, impairment of vascular relaxation, NO reduction, and enhancement of ROS production (Figure 1). The gut microflora derived-p-cresyl sulfate, and its role in vascular disease in CKD, opened the gate to investigate the effect of intestinal bacteria in deriving these uremic toxins. Thus, modification of microflora in kidney disease patients could have a lead potential in reducing the effect of these circulating uremic toxin and their evoked renal fibrosis and CV calamities (Gao and Liu, 2017; Castillo-Rodriguez et al., 2018; Six et al., 2020). This has been the target of some promising preclinical data (Plata et al., 2019; Sato et al., 2020) that is yet to be thoroughly studied, evaluated and applied systematically (Rayego-Mateos and Valdivielso, 2020). Lastly, the aforementioned FGF-23, and its co-receptor klotho have been the focus of investigations of many studies in CKD humans and animals, as those signaling entities play an important role in heart and blood vessel functions and structures (Gutiérrez et al., 2009; Mirza et al., 2009). With worsening CKD, klotho deficiency is becoming a status quo condition, as circulating klotho levels decline, arterial stiffness and hypertension ensue (Gao et al., 2016). Furthermore, a klotho/FGF-23 deregulation state observed in CKD is increasingly becoming linked to resulting CV pathologies such as LVH, vascular calcification, and endothelial dysfunction (Olauson and Larsson, 2013; Erben, 2016).

#### 2.5.5 Effect of sex

Sex differences in CKD appear to play a major but ill-defined role in the incidence, prevalence, and progression of renal disease. Furthermore, our knowledge of the molecular mechanisms governing CV control and renal function in both genders remains limited, which is perhaps, in part, due to the fact that most research studies are primarily conducted in the male gender then findings are generalized to both males and females. Another possible reason for this scarcity of information is that most women with CKD are postmenopausal, which could explain why the influence of female hormones tends to be overlooked. This section therefore provides compelling evidence regarding the importance of sexual dimorphism in investigating pathophysiologic mechanisms relating to CVD in the CKD population.

Clinical studies on CVD in CKD patients suggest that women carry a relatively lower risk of developing CVD and related complications compared with men (Shajahan et al., 2021); however, it remains not entirely understood if these effects, as shown in hypertension, are driven by a differential influence of sex on CV regulatory control mechanisms in CKD. In relation to sex differences in vascular dysfunction, younger women (<55 years old) with CKD appear to show greater endothelium-dependent and -independent relaxation of the brachial artery than men, and that these differences disappear when women’s age exceeds 55 years (Kruse et al., 2020). These data strongly support the notion that vascular dysfunction in women is not as pronounced as it is in men. With RAS, on the other hand, it has been shown that Ang II induces lower increases in BP and proteinuria in female SHRs compared with males. Although the precise mechanisms underlying these differences are incompletely understood, it is believed that estrogens in females may decrease the synthesis of ACE while testosterone in males may promote increased renin release (Quan et al., 2004). Female SHRs also seem to demonstrate greater expression levels of the “*protective*” AT_2_ receptors (Hilliard et al., 2014), and exhibit more profound vasodilatory responses to the AT_2_ agonist Compound 21 (Hilliard et al., 2012). Together, these data, although yet to be replicated in CKD, potentially indicate that male sex is associated with a greater RAS activity compared with females.

In relation to neural control of CV function in CKD, despite many studies reporting autonomic deficits in the CKD population, which contribute to sympathetic hyperactivity and increased CV risk in CKD, there are hardly any reports that dissect study variables based on sex. Our group was the first to show that impaired cardiac and sympathetic baroreflex control in male CKD rats was likely driven by temporal deficits in the functionality of the afferent and central components of the baroreflex arc (Salman et al., 2014), whereas impaired heart rate and sympathetic baroreflex in female rats appeared to result primarily from a sole decline in the central processing of baroreceptor afferent input (Salman et al., 2015a). These results indicate that female CKD rats express fewer autonomic deficits compared with males; however, if similar sex differences in autonomic function can be identified in humans with CKD remains to be determined.

Apart from the role of the vascular-hormonal-neural triad in the development of CVD in CKD, there is now growing evidence supporting a role for inflammation in the development and maintenance of hypertension and renal injury (Caillon and Schiffrin, 2016). It is therefore possible that the pathology of CVD in CKD is also contingent upon sex differences in the expression levels of key inflammatory markers driving the hypertensive state in both sexes. For instance, hypertension is associated with increased renal T cells infiltration (Caillon and Schiffrin, 2016); and the number of T regulatory cells (Tregs), which play a pivotal role in maintaining immune homeostasis via suppressing effector T cells activation (Mahajan et al., 2006) and modulating RAS, is lower in many models of hypertension (Barhoumi et al., 2011; Amador et al., 2014; Fabbiano et al., 2015). Indeed, female SHRs express more renal Tregs, which corresponds with a lower BP and less proteinuria and renal injury (Tipton et al., 2012). However, the role of Tregs in altering the course of CKD progression in both males and females has yet to be addressed in experimental and clinical studies.

## 3 Conclusion

Hypertension, the leading cause of CV incidence derived from and sustained by CKD development and progression, is a crucial challenge for modern medicine, given the rising numbers in morbidity and mortality worldwide. While numerous potential pathophysiological mechanisms have been uncovered in recent years, many details remain poorly understood or unknown, and therefore, continued research and investigation to explore and navigate therapeutic options are imperative in hypertensive kidney disease. Here, we put forward relevant findings which demonstrate mechanisms underpinning raised BP in CKD, while taking into account the existence of multifactorial entities that can initiate or come later into play in the disease process. A deeper understanding of the molecular mechanisms and pathways will allow the identification of key targets of pharmacological or non-pharmacological therapies that are able to achieve an appropriate control of renal damage in hypertension. Consequently, this would give the chance for novel therapeutic strategies to overcome current conventional treatments by their ability to interact with the cardio-renal system. The hormonal, neural, and vascular arms are integral in the regulation of renal and CV physiology, and they amalgamate complex mechanisms, often by interacting with each other, and inadvertently pave the way for the pathogenesis of hypertensive nephropathy. Knowing that, however, more research is required to transfer promising experimental results into routine clinical practice. Although the use of current pharmacological agents targeting systems like the RAAS, SNS, ET-1, voltage-gated Ca^2+^ channels, COX, and renal Na^+^/K^+^/2Cl^−^, ENaC and SGLT_2_ have translated into benefit of use and reductions in CV morbidity and mortality, there remains room for improvement in controlling hypertensive kidney disease, especially when it comes to halting, reversing, or even modulating its adverse action on the CV system.

## Glossary

CKD: Chronic kidney disease
ESRD: end-stage renal disease
CV: cardiovascular
CVD: cardiovascular disease
BP: blood pressure
SNS: sympathetic nervous system
RAAS: renin–angiotensin–aldosterone system
RAS: renin–angiotensin system
GFR: glomerular filtration rate
PKD: polycystic kidney disease
BAT: baroreflex activation therapy
mmHg: millimeter mercury
SPRINT: Systolic BP Intervention Trial
AT1: angiotensin-1 receptor
AT2: angiotensin-2 receptor
ET1: endothelin-1 receptors
Ang II: angiotensin II
SOD: superoxide dismutase
NADPH: nicotine amide dinucleotide phosphate
ADMA: asymmetric dimethylarginine
SDMA: symmetric dimethylarginine
NO: nitric oxide
ROS: reactive oxygen species
LVH: left ventricular hypertrophy
O2•-: superoxide free radical
VSMC: vascular smooth muscle cell
NSAIDs: non-steroidal anti-inflammatory drugs
PGI2: prostacyclin I2
COX: cyclooxygenase
NE: norepinephrine
SNA: sympathetic nerve activity
LPK: Lewis polycystic kidney disease
SHR: spontaneously hypertensive rat
Hz: hertz
PWV: pulse wave velocity
NF-kB: nuclear factor κ‐light‐chain‐enhancer of activated B cells
VCAM-1: vascular adhesion molecule-1
ACEi: angiotensin-converting enzyme inhibitor
MAP: mean arterial pressure
ARB: AT1 receptor antagonist/blocker
EDHF: endothelium-derived hyperpolarization factor
ICAM-1: intracellular adhesion molecule-1
EDRFs: endothelium-derived relaxing factor
EDCFs: endothelium-derived constricting factors
ACh: acetylcholine
M3: muscarinic 3 receptors
Ca2+: calcium
eNOS: endothelial nitric oxide synthase
cGMP: cyclic guanosine monophosphate
l-NAME: Nω-nitro-l-arginine methyl ester
AA: arachidonic acid
TxA2: thromboxane A2
ACE: Angiotensin converting enzyme
ERA: endothelin receptor antagonist
LDL: low-density lipoprotein
TGF-β1: transforming growth factor β1
BMPs: bone morphogenetic proteins
NOS: nitric oxide synthase
L-Arg: l-arginine
nNOS: neuronal nitric oxide synthase
iNOS: inducible nitric oxide synthase
ONOO−: peroxynitrite
AGE: advanced glycation end products
BH4: tetrahydrobiopterin
CAT y+: cationic transporter
DDAH: dimethylarginine dimethylaminohydrolase
PG: prostaglandin
DOCA: deoxycorticosterone acetate
NOARG: nitrogen oxide synthase inhibitor Nω-nitro-l-arginine
SGLT2i: sodium-glucose cotransporter-2 inhibitor
PTH: parathyroid hormone
FGF-23: fibroblast growth factor-23
ENaC: epithelial sodium channels
Na-K-ATPase: sodium-potassium atpase transporter
Tregs: T regulatory cells
